# Antenatal sonographic diagnosis of semilobar holoprosencephaly with associated cleft lip and palate

**DOI:** 10.1259/bjrcr.20180013

**Published:** 2018-10-11

**Authors:** Avinash Naikwadi, Rujuta Rege, Shahul Hameed

**Affiliations:** 1 IQRAA International Hospitals and Research Center, Kozhikode, Kerala; 2 Head of the department, Radiology and Imaging sciences, IQRAA International Hospitals and Research Center, Kozhikode, Kerala

## Abstract

Routine antenatal Level II ( anomaly scan) scanning done in a 36-year-old pregnant patient with no history of birth defects in the previous pregnancy showed a 19 weeks fetus with deformed cerebral ventricular cavities, fusion of thalami and craniofacial abnormalities. Diagnosis of semilobar holoprosencephaly with midline cleft lip-cleft palate was given and termination of the ongoing pregnancy was advised in view of the unfavourable outcome.

## Clinical presentation

A 36-year-old pregnant female was referred for anomaly scan to our department of radiology and imaging sciences. She had two previous pregnancies, both of which were uneventful.

## Imaging findings

**Figure 1.**
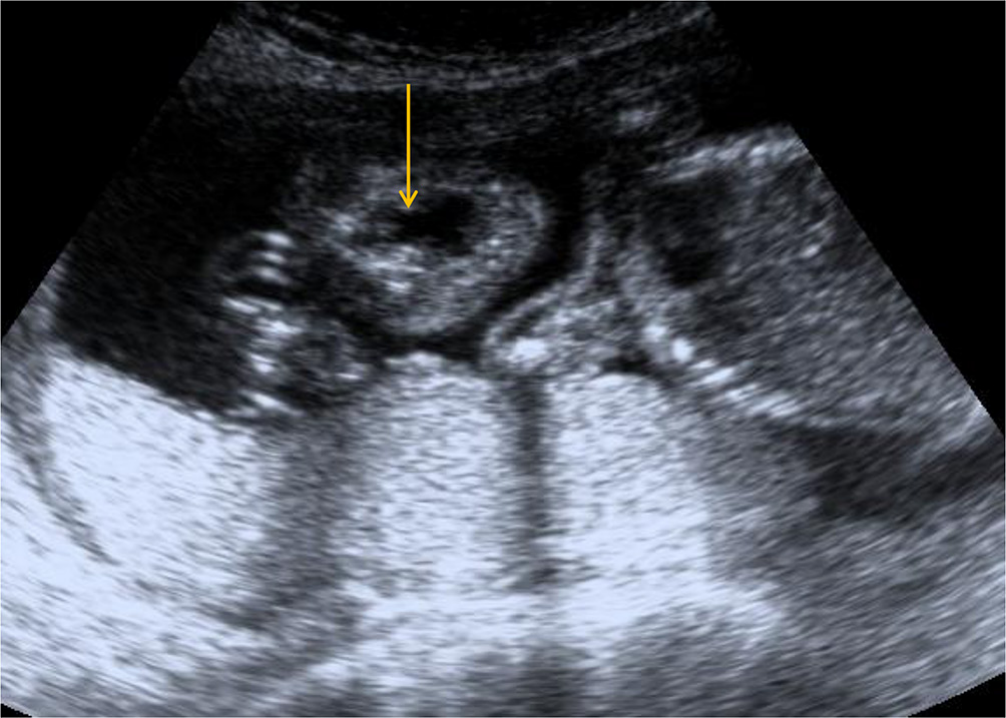
USG image showing hypoechoic midline defect (yellow arrow) in the upper portion of the lip and in the maxilla suggestive of midline cleft lip and cleft palate.

Ultrasound examination of the fetus was done. Premaxillary region of the face was not clearly identified suggesting a possibility of craniofacial malformations. This was seen in the form of a discontinuity, seen as a hypoechoic midline defect in the upper portion of the lip and in the maxilla [Figure 1].

**Figure 2.**
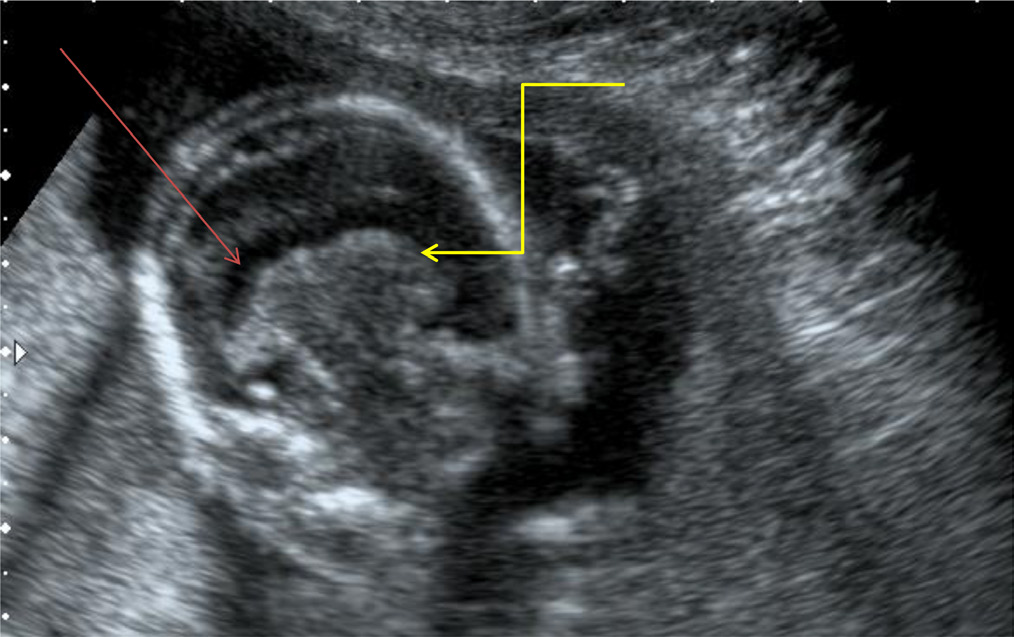
USG showing a single ventricular cavity (red arrow) with complete fusion of thalami (yellow arrow).

**Figure 3.**
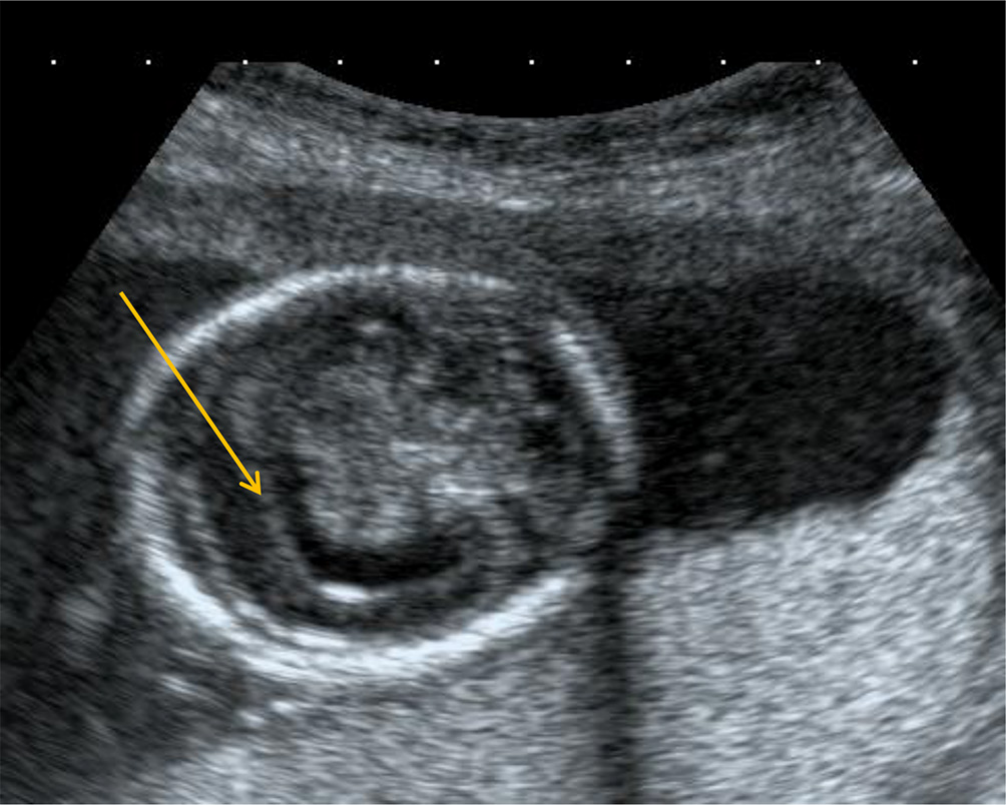
USG demonstrating that there is fusion of the choroid plexus (yellow arrow).

**Figure 4.**
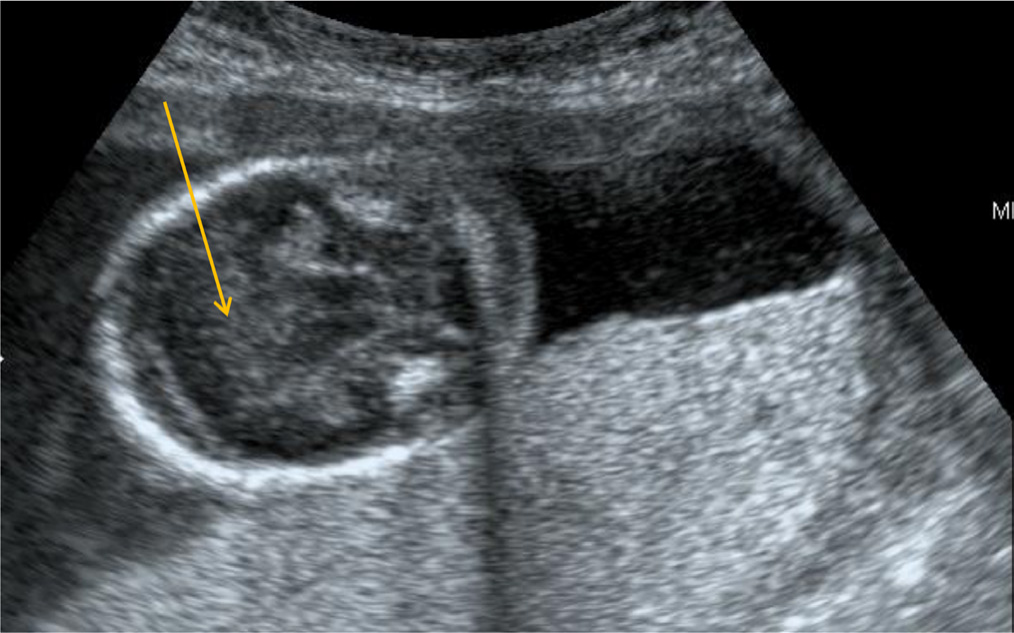
USG showing absence of falx and interhemispheric fissure anteriorly shown by the yellow arrow

Examination of the fetal head showed the presence of a single ventricle and thalami seen in the coronal and saggital views of the brain (Figure 2]). Fusion of the choroid plexus ([Figure 3) and absence of falx was seen anteriorly (Figure 4). Nuchal fold was thickened.

Taking into account the above features, diagnosis of semilobar holoprosencephaly with midline cleft lip-cleft palate was given and the patient was suggested termination of pregnancy.

## Treatment and outcome

**Figure 5.**
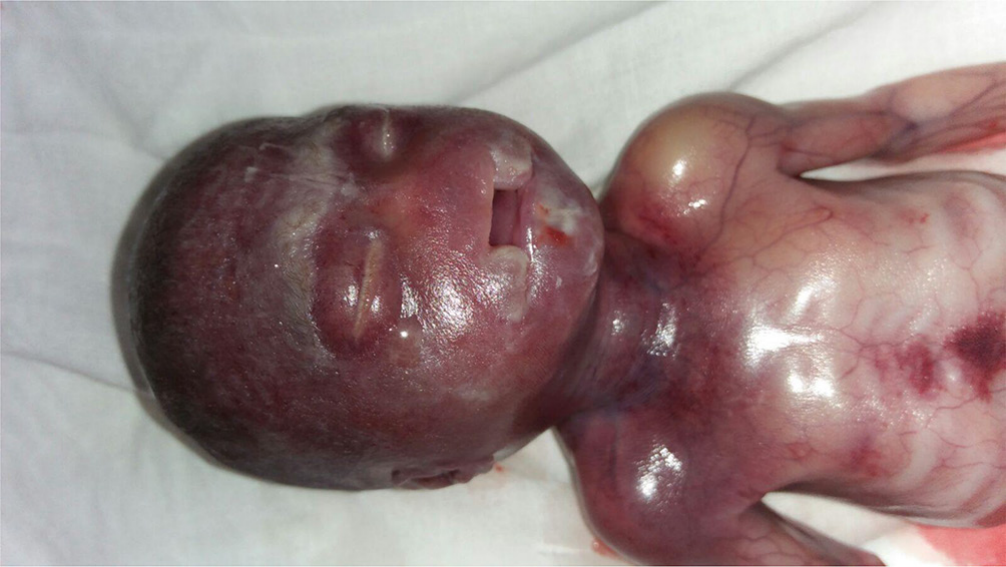
Gross picture of the fetus showing midline cleft lip and cleft palate.

The pregnancy was terminated as the patient was unwilling to proceed further given the severity of the disease and associated poor prognosis beyond the early neonatal life. [Figure 5 showing the gross specimen]

## Discussion

Brain abnormalities are an important cause of childhood morbidity and mortality having an effect on the family in terms of psychological and financial burden.

Classic holoprosencephaly results from a primary defect of ventral induction resulting in a failure to form two separate ventricles during the first trimester.^1,2^ Various malformations exist, which occur due to complete or partial failure of prosencephalic cleavage.

The fetuses are incompatible with life and often lead to spontaneous intrauterine death. The non- classical form of the disease has lesser severity and usually presents with normal or near normal development of the midline structures such as the face, eyes, nose and lips.

### Classification

Depending on the severity, holoprosencephaly is categorized as alobar, semilobar and lobar.^1^

Alobar holoprosencephaly, the most severe form resulting from non-separation of the forebrain into two hemispheres.

- Resulting in a single, unstructured ventricle,
- Unseparated thalami,
- deficiency/non-existence of the corpus callosum, falx, cavum septum pellucidum,optic tracts and olfactory bulbs^3,4^
- Severe facial malformations.
- Semilobar Holoprosencephaly is an intermediary form of the disease.
- Resulting in incomplete separation of the ventricles,
- Partial union of the thalami.^3,4^
- Absence of septum pellucidum.
- Rudimentary falx cerebri.
- Incomplete interhemispheric fissure.
- Agenesis/hypoplasia of corpus callosum.

Lobar Holoprosencephaly, is the least severe form, where there is significant evidence of two brain hemispheres.

### Aetiology and prognosis

Holoprosencephaly occurs periodically and has shown to have a normal karyotype. However, the disorder is shown to have association with trisomy 13, chromosomal deletions and ring chromosome.^1,4–6^

Prognosis depends on the severity of the disease process. Alobar and semilobar holoprosencephaly have worst prognosis resulting in death within the first year of life.^7^ Patients with lobar holoprosencephaly have unpredictable degree of mental, visual and olfactory abnormalities.^8^ However, they have shown to have a normal life expectancy.^6^

## Role of imaging

Since holoprosencephaly has poor survival, prenatal diagnosis plays a significant role in the management and outcome of the pregnancy.

Craniofacial malformations are seen in syndromes which have multisystem involvement,^9^ hence their early recognition is important. Ultrasound is reasonable, dependable and inexpensive in antenatal diagnosis. Defects in the structural development can be identified as early as 11 weeks in the antenatal period.^9^ Assessment of the fetal face can be of a problem in case of maternal obesity, improper position of the fetus, and/or decreased liquor volume. Cranial features are evaluated in axial and coronal planes of the fetal head^10^ while the fetal face is evaluated by mid-saggital, cross-sectional and axial-scans.^10^

### Semilobar holoprosencephaly

Semilobar holoprosencephaly occurs when there is incomplete separation of the ventricles and cerebral hemispheres posteriorly. There is partial division of the thalami, with a single ventricular cavity seen anteriorly.^11^ The incomplete union of the thalami is best appreciated in the coronal plane of the fetal head.^9^ Microcephaly or macrocephaly along with facial defects such as cyclopia, thmocephaly, cebocephaly and midline cleft are some other defects which can occur with semilobar holoprosencephaly.^10^ Hence, it is difficult to differentiate between these two forms of holoprosencephaly but this is not of importance as the next mode of treatment and pregnancy outcome is similar in both.

It is important to differentiate for clinical purpose between alobar and semilobar holoprosencephaly from other causes of large intracranial fluid collections such as severe enlarged ventricles, hydranencephaly, porencephalic cyst and Dandy-Walker cyst, but their differentiation will not affect the fetal outcome. But compared with these abnormalities, holoprosencephaly is associated with an increased risk of chromosomal defects and is shown to have familial tendency. The above mentioned intracranial fluid collections, show presence of midline structures. The supra tentorial ventricular system can be appreciated and characteristic facial features associated with holoprosencephaly are absent. Therefore, accurate analysis of holoprosencephaly is vital for patient counselling and for conducting suitable obstetric management.

## Conclusion

In our case, the pregnancy was terminated as semilobar holoprosencephaly is a fatal form of holoprosencephaly and continuing the pregnancy is traumatic both emotionally and physically for the parent’s hence early prenatal diagnosis plays a crucial role in patient management.

## Learning points

1. Semilobar holoprosencephaly occurs due to mayhem of prosencephalic cleavage. Early imaging and diagnosis helps in deciding the outcome of the ongoing pregnancy.
2. Types of holoprosencephaly can be differentiated with the help of imaging modalities, where ultrasound is the first line modality used and fetal MRI acts as a problem solving tool.
3. Other differentials must be carefully looked for before giving the diagnosis, and screening of the midline structures must be done to rule out other defects like septooptic dysplasia, syntelencephaly etc.

## Consent

Written informed consent was obtained from the patient for the purpose of publication.

## References

[1] Roessler E, Muenke M The molecular genetics of holoprosencephaly. Am J Med Genet C Semin Med Genet 2010; 154C: 52–61. doi: 10.1002/ajmg.c.30236 20104595 PMC2815021

[2] Cohen MM Holoprosencephaly: A mythologic and teratologic distillate. Am J Med Genet C Semin Med Genet 2010; 154C: 8–12. doi: 10.1002/ajmg.c.30252 20082455

[3] Blaas HG, Eik-Nes SH Sonoembryology and early prenatal diagnosis of neural anomalies. Prenat Diagn 2009; 29: 312–25. doi: 10.1002/pd.2170 19194866

[4] Bardo D, Pediatric neuroradiology, part 1: Embryologic basis for brain malformation. Appl Radiol 2009;: 29–40.

[5] Holoprosencephaly DW Elesevier North Holland Biomedical Press. (cyclopia-arhinencephaly) : Vinken PJ, Bruyn GW, Klawans HL. Amsterdam: The British Institute of Radiology.; 1987 225–44.

[6] Babcock DS Sonography of congenital malformations of the brain. Neuroradiology 1986; 28(5-6): 428–439. doi: 10.1007/BF00344097 3540707

[7] Filly RA, Chinn DH, Callen PW Alobar holoprosencephaly: ultrasonographic prenatal diagnosis. Radiology 1984; 151: 455–9. doi: 10.1148/radiology.151.2.6709918 6709918

[8] Chervenak FA, Isaacson G, Hobbins JC, Chitkara U, Tortora M, Berkowitz RL Diagnosis and management of fetal holoprosencephaly. Obstet Gynecol 1985; 66: 322–6. 3895078

[9] Cohen MM An update on the holoprosencephalic disorders. J Pediatr 1982; 101: 865–9. doi: 10.1016/S0022-3476(82)80349-1 7131179

[10] Fowlie A, Holoprosencephaly CG the central nervous system In: Dewbury K, Meire H, Cosgrove D, eds. Ultrasound in obstetrics and gynaecology. Churchill Livingstone; 1993 pp 292–4.

[11] Pilu G, Reece EA, Romero R, Bovicelli L, Hobbins JC Prenatal diagnosis of craniofacial malformations with ultrasonography. Am J Obstet Gynecol 1986; 155: 45–50. doi: 10.1016/0002-9378(86)90075-X 3524242

